# Why have a bottle when you can have draught? Exploring bottle refusal by breastfed babies

**DOI:** 10.1111/mcn.13481

**Published:** 2023-02-03

**Authors:** Clare Maxwell, Valerie Fleming, Lorna Porcellato

**Affiliations:** ^1^ Department of Midwifery, Faculty of Health, School of Public and Allied Health Liverpool John Moores University Liverpool UK

**Keywords:** biopsychosocial model, bottle feeding, bottle refusal, breastfeeding, nipple confusion

## Abstract

Bottle refusal by breastfed babies is a scenario that has received surprisingly little attention in the literature, given the number of mothers who appear to be experiencing it globally and the subsequent negative impact it can have. In line with this, we undertook a study to explore mothers’ views on why their breastfed baby refuses to bottle feed. A parallel, two‐stage, exploratory qualitative design was employed using 30 semi‐structured interviews and 597 online forum posts. Data were analysed using a thematic analysis, and a biopsychosocial model was applied resulting in four overarching themes being identified: ‘Breastfeeding is the answer to everything….’ ‘Bottle feeding: an alien concept… ‘Babies are individuals’ and ‘Find the right bottle and don't delay’. The psychological benefits of breastfeeding, not inherent in bottle feeding, appeared to underpin some mothers’ views on their baby's refusal. Other mothers explained refusal as being down to a baby's biological expectation to be fed by the breast; therefore, bottle feeding was not a normal concept to them. A baby's individual personality and temperament were also suggested as contributing to the scenario and refusal was linked to babies disliking a certain brand of bottle and being introduced to it ‘too late’. This study's findings point to a complex, multifactorial picture underpinning bottle refusal by breastfed babies, which transcends physical, psychological and biological concepts, and is influenced by socio‐cultural norms surrounding infant feeding. Recognition of these contributing factors is needed to aid those supporting mothers experiencing the scenario and, importantly, to underpin mothers’ decision‐making around managing it.

## Key messages

- There is no one definitive reason why a breastfed baby refuses to bottle feed
- The psychological benefits of breastfeeding appear to underpin refusal for some babies.
- Biologically, babies expect to breastfeed; bottle feeding is not a normal concept for them.
- A breastfed baby's individual personality/temperament may underpin their bottle refusal.
- Mothers explain refusal as their baby ‘disliking’ certain bottle brands and being introduced to a bottle ‘too late’.

## 1 INTRODUCTION

The World Health Organization advocates exclusive breastfeeding for 6 months (WHO, 2001); however, it is clear that this goal is not always reached (Victora et al., 2016) and due to psycho‐social, economic or physical reasons, mothers wish to, or may need to, introduce a bottle to their breastfed baby (Gatrell, 2007; Johns et al., 2013; Maxwell et al., 2020; McInnes et al., 2013; Skafida, 2012). For some mothers, however, the transition to bottle feeding, either containing formula or expressed breastmilk (EBM), is not always successful, owing to their breastfed baby's refusal to bottle feed (Maxwell et al., 2020).

Bottle refusal by breastfed babies is a scenario that has received surprisingly little attention in the literature, given the number of mothers globally who appear to be experiencing it (Maxwell et al., 2020). Hundreds of thousands of references are made to it in global breastfeeding groups, parenting forums and on social media with an emphasis on the negative impact of bottle refusal and, in turn, requests for advice on how to ‘solve it’.

There is no formal definition of what constitutes bottle refusal by a breastfed baby. Previous references have centred around it being a form of ‘nipple confusion’: a term mainly used to describe a breastfed baby who, when introduced to bottle feeding, becomes ‘confused’ due to the two feeding mechanisms being different and therefore gravitates towards bottle feeding. (Batista et al., 2019; Neifert et al., 1995). However, Neifert et al. (1995) also describe another type of nipple confusion, described as ‘when an older infant who is proficient at breastfeeding refuses to drink from a bottle’ (p.128). A more recent and complete definition of bottle refusal by breastfed babies was developed by the authors of this paper, taking into account the nuances surrounding the scenario. We define the scenario as ‘when a breastfed baby initially or continuously refuses to accept a bottle containing either expressed breastmilk or infant formula’ (Maxwell et al., 2020).

Alternatives to a bottle do exist. Cup feeding has been found to have benefits in terms of increased breastfeeding duration when compared with a bottle in the preterm population (Allen et al., 2021). However, cup feeding appears to be unpopular with mothers and has itself been reported as being refused (Maxwell et al., 2020). In addition, there have been concerns over adequate intake, spillage (Collins et al., 2004) and noncompliance by mothers (Flint et al., 2016).

The impact of bottle refusal by breastfed babies can be acutely negative, with mothers reporting delaying their return to work, feeling isolated and depressed, and being ‘forced’ to breastfeed rather than wanting to. In some instances, mothers have reported having to cancel or delay emergency surgery (Maxwell et al., 2020). Critically, Maxwell et al. (2020) found that over a quarter of mothers (*n* = 210) experiencing bottle refusal by their breastfed baby reported that it had a negative impact on their breastfeeding experience, which could have detrimental consequences for future breastfeeding decisions and practices.

Managing bottle refusal by breastfed babies, including advice and support giving, is challenging, given that the reasons for a baby's refusal are largely unknown. This is of concern, as mothers have previously reported resorting to unevidenced and anecdotal methods to ‘solve’ their baby's refusal, which can damage both their own and their baby's health (Maxwell et al., 2020). Examples include mothers employing ‘cold turkey’—not breastfeeding their baby until it accepts a bottle—which can lead to mastitis or breast abscess in the mother and dehydration in the baby. In addition, mothers have reported sweetening the teat/bottle (Maxwell et al., 2020).

A study was undertaken by the current authors to explore mothers’ experiences of bottle refusal by their breastfed babies to generate knowledge and recognition of the scenario. This paper will focus on one of the study's central research questions: Why do breastfed babies refuse to bottle feed? Mothers’ perspectives on this were explored to provide infant‐feeding personnel and mothers experiencing bottle refusal with an understanding of potential reasons underpinning refusal. This in turn can guide advice, and support and aid mothers’ subsequent decision‐making around the management of the scenario.

## 2 METHODS

### 2.1 Design

A parallel, two‐stage, exploratory qualitative design was employed using semistructured interviews and online forum posts. Data were analysed separately for the interviews and posts and then integrated to provide overarching findings.

#### 2.1.1 Semi‐structured interviews

Semi‐structured interviews were undertaken by the lead author with a sample of UK mothers (*N* = 30) who had initially completed an online survey concerning bottle refusal by their breastfed baby (Maxwell et al., 2020). The inclusion criteria for the survey comprised UK mothers who had experienced bottle refusal by their breastfed baby in the past 5 years or who were experiencing it at the time of completion. The survey was posted online on breastfeeding Facebook groups and UK parenting forums and was completed by 841 UK mothers (Maxwell et al., 2020). Mothers who were interested in being interviewed were asked to leave their details at the end of the survey. Due to 354 mothers expressing an interest in being interviewed, case selection was undertaken based on a simple, maximal variation sampling framework as described by Gray (2014). As the demographic profile of the mothers completing the initial online survey was narrow in terms of age, ethnicity and education, the sampling framework was based on mothers’ differing experiences and outcomes of bottle refusal information, which had been captured from the survey. Fifty‐four mothers were invited for interview, with 30 eventually participating (see Table 1 for participant characteristics and interview details).

**Table 1:** Interview sample: semi‐structured interviews.

Id	Interview mode	Interview length (min)	Impact of bottle refusal on breastfeeding experience	Employment status, age, ethnicity	Eventually accepted bottle?	Breastfeeding duration
1	Face‐to‐face pilot	53	Positive	1–3, 30–34, White	Yes	Stopped 9 months
2	Face‐to‐face pilot	52	No impact	4–6, 30–34, White	No	Still feeding 4 months
3	Face‐to‐face	58	Negative	Student, 25–29, White	No	Still feeding 2.5 years
4	Phone	100	Positive	1–3, 30–34, white	Yes	Stopped 13 months
5	Phone	44	No impact	1–3, 30–34, White	No	Still feeding 22 months
6	Phone	58	Positive	LAF^a^, 35–39, White	No	Still feeding 14 months
7	FaceTime	57	Negative	SE^b^, 30–34, White	No	Still feeding 6 months
8	FaceTime	53	Other	1–3, 25–29, White	No	Still feeding 6 months
9	Phone	48	Negative	LAF, 30–34, white	No	Still feeding 6 months
10	Face‐to‐face	64	Negative	1–3, 35–39, White	Yes	Stopped 15 months
11	SKYPE	59	No impact	LAF^a^, 35–39, White	No	Still feeding 10 months
12	SKYPE	104	Negative	1–3, 30–34, Mixed	No	Still feeding 10 months
13	Phone	101	Positive	1–3, 30–34, White	No	Still feeding 4 months
14	Phone	58	No impact	1–3, 25–29, White	Yes	Stopped 3 years
15	Phone	52	No impact	LAF^a^, 30–34, White	Yes	Stopped 7 months
16	Phone	42	No impact	1–3, 35–39, White	No	Still feeding 10 months
17	SKYPE	71	Negative	1–3, 30–34, White	Yes	Stopped 1 year
18	Phone	52 Partial	No impact	4–6, 30–34, White	Yes	Still feeding 4 months
19	Phone	45	No impact	1–3, 30–34, White	No	Still feeding 6.5 months
20	SKYPE	52	Negative	1–3, 35–39, White	No	Still feeding 11 months
21	FaceTime	45	No impact	LAF^a^, 30–34, White	No	Still feeding 9 months
22	Phone	64	Negative	4–6, 25–29, White	Yes	Still feeding 13 months
23	Face‐to face	59	Negative	1–3, 30–34, White	No	Still feeding 4 months
24	Phone	46	Negative	1–3, 30–34, White	yes	Stopped 15 months
25	Phone	52	Negative	1–3, 35–39, White	No	Still feeding 7 months
26	Phone	50	Negative	1–3, 30–34, White	No	Still feeding 9 months
27	Phone	45	Positive	LAF, 30–34, White	No	Still feeding 1 year
28	Face‐to‐face	50	Positive	1–3, 30–34, White	No	Still feeding 7.5 months
29	Phone	48	No impact	1–3, 25–29, White	Yes	Stopped 11 months
30	Phone	46	Negative	1–3, 30–34, White	Yes	Not known
31	Face‐to‐face	42	Positive	1–3, 35–39, White	No	Still feeding 13 months
32	Face‐to‐face	140	Positive	1–3, 40+, White	No	Still feeding 1 year

^a^ Looking after family.

^b^ Self‐employed UK Office for National Statistics Categories 1–3: Managers, directors, senior officials, professional occupations, associate professional and technical; 4–6: Administrative and secretarial, skilled trades, caring, leisure and service; 7–9: Sales and customer service, process, plant and machine operatives, elementary occupations.

Mothers were offered five modes of interview: in person if they lived locally to the author, SKYPE, FaceTime, WhatsApp or by phone. This decision was primarily taken to reduce ‘participant burden’ (Daniels et al., 2012; p.2) and increase recruitment. An interview schedule was developed in line with results from the online questionnaire in relation to previous literature, and in consultation with experts in the field of infant feeding. A pilot study was undertaken with two mothers who had experienced bottle refusal with minimal changes being made to reduce the overlap of questions. Questions to explore why breastfed babies refuse to bottle feed included ‘How does your baby react when offered a bottle? Why do you think they react in this way? Why do you think your baby refuses to bottle feed? What could you have done to prevent bottle refusal?’ All mothers interviewed were allocated an ID number, for example, ID 29 to ensure confidentiality and anonymity. Consent was gained at the beginning of each interview either via hard copy (face‐to‐face) or verbally (nonface‐to‐face) with the latter being digitally recorded. Interviews were digitally recorded and took place between April and June 2017 ranging from 42 to 140 min. Interviews were transcribed verbatim by the lead author and imported directly into NVivo 11 for analysis (QSR, 2018). Data saturation, whereby no new themes were identified (Bryman, 2016; Saunders et al., 2018) was achieved after 30 interviews. Braun and Clarke (2019) discuss data saturation as not being consistent with reflexive thematic analysis, particularly in terms of agreed numbers of data items to be collected. In acknowledgement of this, our number of interviews was never ‘predefined’, which we believe allowed us to flexibly and comprehensively collect data with a view to answering our study aim.

#### 2.1.2 Online forum posts

Individual online forum posts around bottle refusal by breastfed babies were captured retrospectively from MUMSNET.com, Netmums.com and Babycentre.co.uk (all UK‐based parenting forums) over a 3‐month period between March and June 2017 (*N* = 597). This enabled mother to mother discussions around the scenario to be explored. Although it was not possible to capture individual demographic data from the posters, forum analytics showed that they were most likely UK‐based. Inclusion criteria comprised threads and posts that were in the public domain, did not require membership to view and pertained to bottle refusal by breastfed babies using the keywords ‘bottle refusal’, ‘breastfeeding’ and the abbreviation ‘bf’. Appropriate discussion boards were selected, and threads were searched for using the keywords. Posts were then captured within the threads. To ensure confidentiality and anonymity was preserved, posts were allocated ID codes comprising of the thread number and the initials of the forum they were posting on, that is, T7 nm = thread 7 netmums, T7 mn = thread 7 mumsnet and T4 bc = thread 4 babycentre. Posts were captured from the forum sites using NCapture and imported directly into NVivo 11 for analysis.

### 2.2 Data analysis

To elucidate the nuances of breastfed babies’ refusal to bottle feed, a thematic analysis and a biopsychosocial model of health were used—the latter first being proposed by Engel (1977). This model was developed in response to an emphasis on a reductionist, dualistic, biomedical model of health, which separated body from mind by giving credence to the subjective, contextual experience of patients and the impact this has on their health (Borrell‐Carrio et al., 2004). The use of a biopsychosocial model has been applied previously to understand children's eating (Berlin et al., 2009), and disordered eating and breastfeeding among postpartum mothers (Rodgers et al., 2018). The model is useful in understanding why breastfed babies refuse to bottle feed due to its appreciation of the interplay between the sociocultural, psycho‐emotional and biological influences on breastfeeding. It also recognises the multiple stimuli associated with infant development rather than defaulting to a simplistic, biological interpretation (Newman et al., 2015).

A six‐stage thematic analysis was employed using Braun and Clark's (2013) approach rather than their revised one (Braun & Clarke, 2019), as analysis of the individual data sets had commenced before its publication. This analysis comprised familiarisation with the data, coding across the data, generating initial themes, review of initial themes, refining, defining and naming themes, and writing up the findings (Braun & Clark, 2013). During the thematic analysis of the interview data and online posts, the concepts of the biopsychosocial model were also drawn upon. The model was used to guide coding and theming, ensuring codes and themes outside of the model could also be identified, thus resulting in both an inductive and deductive process being undertaken. To ensure analytical rigour, a colleague outside of the research team, familiar with thematic analysis, undertook blind coding of part of one of the transcribed interviews and a sample of the online posts across all three forums. Codes were then checked against the original codes for similarity. The colleague also reviewed a subset of the codes and corresponding themes to ensure they were credibly linked. Only minor suggestions were made due to a high similarity index being found during both exercises. Three themes were identified from the interviews and three from the online posts.

Final integration of the two sets of findings was undertaken using a narrative approach of ‘weaving’ as described by Fetters et al. (2013). Weaving occurs by the findings of multiple data sets being ‘woven’ together around a ‘central concept’ to produce overall merged findings. Fetters et al. (2013) do not provide a step‐by‐step guide in terms of the weaving process. It was therefore decided to employ a systematic approach to the integration not unlike Braun and Clarke's previously used thematic analysis to ensure rigour. First, the data codes and themes were reread to aid refamiliarisation. Next, the six themes and corresponding codes were brought together in tabular form to begin a preliminary visual integration. Then, weaving of the themes and codes was undertaken using the research question ‘Why do breastfed babies refuse to bottle feed?’ as the central concept. During this stage, the biopsychosocial model was referred to, in order to provide further focus to the weaving of the data. Finally, the weaving process was complete with four, new, overarching themes being developed, which were subsequently named ‘Breastfeeding is the answer to everything’, ‘Bottle feeding: an alien concept’, ‘Babies are individuals’ and ‘Find the right bottle and don't delay’.

### 2.3 Ethics

Full ethical approval was gained from the Liverpool John Moores University ethics committee.

## 3 FINDINGS

The demographic profile of the mothers was analysed using UK Office for National Statistics (ONS) categories, which provide categories including employment and ethnicity for use in statistical analysis. The majority of mothers who were interviewed were White, employed in ONS Groups 1–3 (ONS, 2016) and aged over 30 years (see Table 1). According to the last UK infant‐feeding survey (McAndrew et al., 2012), the latter two demographics are representative of mothers most likely to breastfeed in the United Kingdom, although this association should be treated with some caution given that the survey is now over 20 years old. The mothers were atypical, however, in terms of the duration of their breastfeeding, (ranged from 4 months to 2.5 years), with the majority of UK mothers having given up breastfeeding exclusively by 6 weeks and 96% having introduced a bottle by this time (WBTi, 2016; Gov.co.uk, 2022). Although it is not possible to authenticate the demographics of the mothers who posted on the forums, analytics show the majority of users are educated to graduate level (college/university), female and from the United Kingdom (Similarweb.com).

### 3.1 Breastfeeding is the answer to everything

‘Breastfeeding is the answer to everything’ emerged from discussions and posts surrounding the non‐nutritional attributes of breastfeeding, which were underpinned psychologically. These properties were perceived to be all‐encompassing to babies and, critically, were not viewed as being available when bottle feeding. Mothers described breastfeeding as a ‘comfort’ and ‘quick fix’ if a baby was upset or tired. It pacified babies and was referred to as the ‘answer to everything’,

*It was just, kind of…it is amazing. It is fantastic how breastfeeding just seemed to sort every problem out* (Id 29).

Due to the frequent and close proximity to a mother that breastfeeding afforded a baby, breastfeeding was often portrayed as a conduit in terms of gaining a mother's physical presence. Interestingly, the period of contact required was often short rather than prolonged, with babies appearing to ‘check in’ with their mother,

*I pick him up it's almost an instant calming effect and it's a very symbiotic relationship…. it's not even that they are hungry it's that they have got to the point that they need to reconnect with the mum. Sometimes he will be crying and I'll think ‘oh he must be really hungry’ and he'll have the tiniest little feed and then he'll be happy again and you think ‘oh he just wanted that little bit of comfort and reassurance’* (Id 9).

In line with this, some mothers described bottle refusal being about babies making a preference for their mother,

*I think he had chosen me over the bottle* (Id 4).

*It's not the bottle that's putting your DD [dear daughter] off ‐ it's just because it's not you and she knows what's nicer!!!* (T7 nm).

In addition, for some babies, breastfeeding was described in terms of attachment to their mother, providing what could be considered as a ‘secure base’,

*Well you see X, he didn't have an attachment with anything, he never had a dummy, he never had a blanket, he never had a particular toy that he was interested in, so I think I was his comfort, I was providing everything he needed, he didn't need anything externally* (Id 4).

The majority of mothers in this study saw bottle feeding as a form of nutrition only, thus restricting it to a physical function rather than a psychosocial or emotional one. Bottle feeding was unable to provide babies with the non‐nutritional benefits that breastfeeding could which was viewed by some mothers as an underlying cause of bottle refusal,

*Interviewee: I think in an ideal world to look on it as a combination of both [bottle and breast], so your partner could feed it ‐ but then I think you are just looking at it purely from a feeding perspective just to get food into them and that's not what breastfeeding is all about*.

*Interviewer: What is it about?*

*Interviewee: It's the bonding, it's the benefit to the baby, if we were only interested in nourishment then there would be no bottle refusal would there?* (Id 11).

### 3.2 Bottle feeding: An alien concept

The theme ‘Bottle feeding: an alien concept’ encompasses mothers’ posts and discussions surrounding bottle feeding not being a baby's ‘biological norm’, with this being reserved for breastfeeding. Feeding from a bottle was not portrayed as a natural concept when compared to breastfeeding and was thus considered a reason for refusal,

*I just think it's this alien concept that there is this thing in her mouth that's not a nipple* (Id 4).

The ‘unnatural’ connotations assigned to bottle feeding were also represented in polarised comparisons between a bottle and breast. Mothers often described the shape and texture of the bottle and teat [artificial nipple], the ‘cold, hard, plastic teat’ being compared unfavourably to the ‘warm, soft, breast’. This was often represented in mothers’ descriptions of their baby's negative physical reactions to being introduced to a bottle,

*She wouldn't even have it in her mouth [bottle teat]. She absolutely hated it…*(Id 2).

Of interest were discussions around bottle refusal, which also encompassed dummy (pacifier) refusal, with a baby's rejection of both being similarly based on the physical nature of a teat.

In line with the disparities between teat and breast, the different feeding mechanisms of bottle and breastfeeding were also described by mothers as a reason why their baby refused to bottle feed. This was not portrayed in the same light as outright refusal or rejection, but more in terms of a skill or object that their baby did not understand or recognise and thus could not master,

*He just doesn't know what to do at all he just can't make it function and he just doesn't understand* (Id 23).

Some mothers highlighted their baby's expectation that milk should/would be provided via breastfeeding only and when it wasn't refusal ensued. This was perceived as being a natural and in most cases not unreasonable expectation by their baby,

*He (husband) tried a bottle and sippy cup and she was not impressed one bit…she looked disgusted that he'd even attempt to give her milk in anyway other than from source* 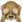
(T2 nm).

*Eventually he would take formula from a bottle, he would not take expressed breastmilk from a bottle, it was like ‘sorry, this is a mismatch’ this is not right* (Id 4).

In addition, there was a recognition of breastfeeding being the ‘obvious’ mechanism of infant feeding for a baby, which mothers would illustrate in terms of breastfeeding's ‘superiority’ over bottle feeding,

*Why have a bottle when you can have draught?* 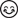 (T7 mn).

### 3.3 Babies are individuals

The theme ‘Babies are individuals’ emerged from mothers’ discussions and posts that referred to bottle refusal being driven by a baby's individual personality, behaviour and/or temperament, aspects that are intrinsic in nature and thus likely to be ‘non‐modifiable’. They represented a further psychological constituent to why babies refuse to bottle feed. A number of mothers described their babies as ‘knowing what they want’ in relation to breast over bottle and often couched this in terms of their baby's determination,

*She would not give up. She would not back down* (Id 19).

In addition, mothers often attributed strong, individual, characteristics to their baby's personality and linked this to their refusal,

*He always has been quite headstrong and knows what he wants to do. I wouldn't be surprised if someone found a link between this and what they were like as a child* (Id 9).

The road to eventual bottle acceptance, particularly when mothers refrained from breastfeeding until their baby eventually accepted a bottle, known as ‘cold turkey’, was often presented as adversarial between baby and mother,

*It took 48 h of constant refusal and strops (on her part) but finally she took a bottle early Mon morning* (T3 nm).

To give further credibility to babies’ individualistic behaviour underpinning bottle refusal, mothers gave various examples of their baby's actions, which were sometimes inexplicable and unpredictable. For some babies who did eventually accept a bottle, this was only from a certain individual,

*Now she will take the occasional bottle of formula from my sister but not from her dad* (T3 mn).

Other babies would only accept a bottle at certain times of the day, had initially accepted a bottle but then suddenly refused it, or, as previously depicted, would only accept formula, not EBM, in a bottle. This unpredictable behaviour was highlighted by mothers whose baby eventually accepted a bottle, although they could not always pinpoint as to why this had occurred,

*He suddenly just took it, I did nothing different … to this day I still don't know why* (Id 24).

Although some mothers raised concerns about there being something ‘wrong’ with their baby due to their refusal to bottle feed, others gave greater credence to babies as individuals, something that isn't always recognised,

*I think we don't allow people enough to acknowledge the differences between babies* (Id 17).

### 3.4 Find the right bottle and don't delay

The theme ‘Find the right bottle and don't delay’ emerged from mothers’ discussions around ‘solving’ bottle refusal and also preventing it with future babies. There was a strong focus, particularly within the online forums, on bottle refusal being a consequence of a baby disliking the bottle type/brand that the mother was using. This may have been derived from the inherent socio‐cultural norm of bottle feeding in the United Kingdom. Advice giving within online forums was prolific and often paramount to advertising, with mothers citing bottle brands that had ‘worked’ (led to bottle acceptance), although this was often described as a lengthy and costly process,

*We tried Tommee Tippee, MAM, NUK, AVENT, the medela ones that come with pump until someone suggested the minibe, she wolfed it down with that* (T4 bc).

No one bottle brand appeared to be more successful than another, and what worked for one baby did not always work for another. In addition, very few mothers advised ‘sticking to one bottle’, indicating refusal was more about the brand of bottle rather than a bottle per se. This was further evidenced in some bottle brands being considered particularly effective for bottle refusal,

*I'm considering a minbie bottle, they're meant to be good for bottle refusers!* (T13 bc).

along with others that were promoted as being ‘akin to breastfeeding’,

*We tried tommee tippee first and she wasn't keen. Then tried Lanisoh which are supposed to replicate the boob in that milk will only flow if she latches and sucks. She loves these and guzzles it down* (T6 bc).

Timing of the introduction of a bottle was seen by most mothers as critical in preventing bottle refusal. Many described how they had ‘left it too late’ and that not introducing a bottle ‘early’ had led to refusal,

*I really wish I'd done it sooner, a lot of the mums I've spoken to who have successfully managed to breast and bottle feed all did it early on* (Id 5).

The reason for delaying the introduction of a bottle was almost exclusively affiliated with advice from health professionals that this prevented nipple confusion, with no mothers discussing or posting about the potential negative impact bottle feeding can have on the establishment of milk supply and breastfeeding per se. Mothers described guidance being to establish breastfeeding first, and to only introduce a bottle around the 6 weeks mark, with no discussion of potential bottle refusal as an outcome of this,

Hindsight is a wonderful thing but after having my eldest I realised the advice I was given by the midwife & HV [Health Visitor] to wait until my son was 6 weeks old before introducing a bottle to avoid “nipple confusion” was an utter load of *#% (insert word of choice!) (T7 nm).

Due to their belief that delaying giving a bottle to prevent nipple confusion had contributed to their baby's bottle refusal, some mothers discussed early introduction with their next baby and advised others to do the same.

## 4 DISCUSSION

This study aims to explore why breastfed babies refuse to bottle feed through mothers’ views. Our findings point to a complex, multifactorial picture, which encompasses physical, psychological and biological concepts underpinning refusal. This is influenced by socio‐cultural norms surrounding infant feeding. In addition, it is evident that reasons for refusal embody both intrinsic, nonmodifiable factors such as baby temperament and personality, and extrinsic modifiable factors such as bottle brand and timing of introduction.

Breastfeeding exhibits non‐nutritional and resultant psychological properties for both infants and their mothers (Linde et al., 2020), and this appeared to reverberate with many mothers in our study. Breastfeeding was seen as ‘all‐encompassing’ and ultimately enabled babies to make a connection with them as mothers. Psychological dependence on breastfeeding was evidently exhibited by some babies in our study, behaviour which could be assimilated with the (albeit older) children and toddlers in Gribble's (2009) study, who described breastfeeding when they were hurt, upset or tired, using it as a way of being ‘close to mummy’ (p.1072).

When compared with bottle feeding, breastfeeding ensures greater physical proximity and more contact time between mother and baby (Smith & Forrester, 2017) and has been found to increase mother–baby communication (Shloim et al., 2015, 2017) and provide pain relief after young babies’ vaccinations (Harrison et al., 2016). Thus, one could propose that bottle refusal is influenced by a baby's preference to gain/retain the non‐nutritional rewards that are associated with breastfeeding which indicates breastfeeding being the secondary rather than primary driver. In line with this, the potential psychological ‘deficits’ of bottle feeding in comparison with breastfeeding are now recognised, with ‘responsive bottle feeding’ being advocated which encourages caregivers to develop a close and loving relationship with their baby (www.unicef.org.uk).

Physiologically, breastfeeding rather than bottle feeding is the ‘biological norm’ in terms of infant feeding. Term, healthy babies are biologically prepared to breastfeed from birth and can ‘self‐attach’ to the breast at birth using preconditioned reflexes (Yin et al., 2021). Mothers in our study highlighted the ‘unnaturalness’ of bottle feeding to their baby, both in terms of its physicality and mechanism, and that there was an ‘expectation’ from a baby to receive milk via the breast. For these babies, one could deduce that the biological norm to breastfeed is a key component in refusal and bottle feeding is indeed an ‘alien concept’.

The mechanics of bottle and breastfeeding have long been viewed as being distinctive from one another (Woolridge, 1986) and these differences were highlighted by mothers in our study as underpinning bottle refusal in that their baby did not know ‘how to’ bottle feed. However, Kotowski et al. (2020) integrative literature review found similar use of tongue and jaw movements between breast and bottle feeding infants, and that differences were due to characteristics of bottles and teats, and caregiver interaction rather than the mechanisms of the two feeding methods. Furthermore, mothers in our study described other babies they knew being able to feed indiscriminately from both breast and bottle. Thus, although bottle refusal was mooted in our study as being due to babies being unable to comprehend bottle feeding, this is not the case for all.

The potential link between ‘dummy refusal’ and bottle refusal provides an intriguing insight into bottle refusal being attributed to physical sensation for some babies. Childhood rejection of foods owing to a heightened sensitivity to taste and texture is suggested by Russell and Worsley (2013), and Cappellotto and Olsen (2021), and a recent study by Ustun et al. (2022) has shown that the fetus can respond negatively to certain tastes while in utero. The fact that mothers in our study reported their baby refusing both expressed breastmilk and formula in a bottle and cup indicates that ‘taste’ does not appear to be implicated in refusal. However, the differences in texture of an artificial teat versus a mother's nipple could well be implicated.

Mothers in our study made links between their baby's personality and individuality, and bottle refusal evidencing a further underpinning psychological element to the scenario. Few studies have explored weaning from breastfeeding and how baby personality impacts this, with those that have, being undertaken with older infants. Marquis et al.'s (1998) study of breastfeeding children in Lima found that those children classed as ‘demanding’ and ‘strong willed’ were able to maintain their breastfeeding status despite maternal wishes to wean them. It could be postulated that babies in our study wanted to breastfeed, to retain a locus of control and agency in terms of their feeding: a concept acknowledged by mothers in Burton et al.'s (2022) study on extended breastfeeding. Due to the intrinsic nature of personality and temperament, bottle refusal in these instances would be difficult to manage with change most likely being ‘baby led’.

Interestingly, none of the mothers in our study implicated their own management of bottle refusal as being a contributor to the scenario. Associations between children's rejection of foods and maternal/parental pressure to eat are well‐documented (DeCosta et al., 2017; Scaglioni et al., 2018), and when extricating these associations to bottle refusal, pressure to bottle feed and a baby associating this with a negative experience are plausible reasons for rejection.

Delayed introduction of a bottle to a breastfed baby was highlighted as a contributory factor to bottle refusal in our study, and being an extrinsic factor it is likely to be modifiable. This points to an emphasis on timing and routine, factors very much linked to the socio‐cultural narrative around infant feeding in high‐income countries where a more technical and medicalised model prevails (Dykes, 2005; Faircloth, 2010; Stearns, 2013). However, the link between timing and bottle refusal is fraught with complexity in that what mothers constitute as ‘early’ and ‘delayed’ introduction is difficult to define. In addition, Maxwell et al. (2020) in their online survey investigating bottle refusal found that babies who eventually accepted a bottle were significantly older at the first attempt of introduction than babies who continued to refuse (*mdn* 8 v 12 weeks, *p* ≤ 0.001). Further to this, delayed introduction of a bottle was often attributed to prevention of nipple confusion and subsequent detrimental effect on breastfeeding duration. However, the evidence surrounding nipple confusion is inconclusive in terms of ‘causality’, as highlighted by Zimmerman and Thompson (2015) who state, ‘The primary difficulty’ is ‘determining whether bottles’/pacifiers’ nipples are causing infants to refuse the breast or whether they are simply markers of other maternal/infant characteristics.’ Interestingly, no mothers in our study made the connection between bottle feeding and reduced milk supply, which physiologically can have an impact on breastfeeding (O'Connor et al., 2018).

Given the mass marketing of bottles and teats specifically to mothers who are breastfeeding (Medela.com, Mimijumi.com, Tommeetippee.co.uk), and in some cases marketed for bottle refusal per se (Minibe.co.uk), it is unsurprising that mothers in our study described bottle refusal as being due to a ‘mismatch’ between bottle and baby. This was particularly evident within the forum discussions where examples depicted babies only accepting a certain bottle brand. However, using different bottles and teats in an attempt to ‘solve’ bottle refusal has been found to have a low success rate with only 15% of mothers who completed an online questionnaire reporting this as leading to eventual acceptance (Maxwell et al., 2020).

### 4.1 Strengths and limitations

This study is not without its limitations. The interview sample was recruited from a larger convenience sample and, although an attempt was made to vary the sample of mothers, the end sample primarily comprised White, older mothers employed in ONS Categories 1–3. Analytics captured by the forums indicate posters are well‐educated, likely aligning them to the employment categories of the mothers who were interviewed. According to the last, albeit dated, UK infant‐feeding survey (McAndrew et al., 2012), these characteristics reflect mothers who breastfeed in the United Kingdom; however, the sample excludes the voices of mothers from ethnic minority groups—the mothers most likely to breastfeed in the United Kingdom. According to the inclusion criteria for the original study and thus subsequent interviews, some of the mothers could have experienced bottle refusal up to 5 years ago, which may have affected memory recall. In addition, data captured from forum posts are difficult to authenticate. Strengths lie in the uniqueness of the study, which, to our knowledge, is the first of its kind to explore why breastfed babies refuse to bottle feed, making an important contribution to infant‐feeding literature and practice.

## 5 CONCLUSION

Our study presents a thought‐provoking insight into the complexities of infant‐feeding behaviour opening up a new debate concerning why some breastfed babies refuse to bottle feed. Study findings point to the biopsychosocial influences on infant feeding, which, in turn, provide potential understanding around the scenario. This knowledge can be translated into guidance for infant‐feeding personnel and for mothers themselves in terms of their management and decision‐making surrounding bottle refusal by breastfed babies. Crucially, this study also adds to the evidence that babies are active participants in infant feeding, and that this is ‘something they do, rather than something that is done to them’ (Rapley, 2015).

## AUTHOR CONTRIBUTIONS

Clare Maxwell performed the research. Clare Maxwell, Valerie Fleming and Lorna Porcellato designed the research study. Clare Maxwell, Valerie Fleming and Lorna Porcellato wrote the paper.

## CONFLICT OF INTEREST STATEMENT

The authors declare no conflict of interest.

## Data Availability

Data is available on request.
